# Development and validation of a drug clinical trial participation feelings questionnaire for cancer patients

**DOI:** 10.3389/fphar.2024.1371811

**Published:** 2024-06-18

**Authors:** Chaowei Guo, Shujun Xing, Guo Zhao, Dawei Wu, Ning Li, Shuhang Wang, Ling Yu

**Affiliations:** ^1^ Department of Community Nursing, School of Nursing, China Medical University, Shenyang, Liaoning, China; ^2^ Clinical Trial Center, National Cancer Center/National Clinical Research Center for Cancer/Cancer Hospital, Chinese Academy of Medical Sciences and Peking Union Medical College, Beijing, China; ^3^ Phase I Clinical Trails Center, The First Affiliated Hospital of China Medical University, Shenyang, Liaoning, China

**Keywords:** cancer nursing, clinical trials, nursing, education, participation feelings

## Abstract

**Objective:**

The study was designed to develop and validate a new drug clinical trial participation feelings questionnaire (DCTPFQ) for cancer patients.

**Methods:**

Data collection and analysis involved a combination of qualitative and quantitative methods. There were two phases to this study. Phase Ⅰ involved developing a questionnaire to establish a list of items to be included in the pool: A theoretical framework was constructed based on the transitions theory and the Roper–Logan–Tierney theory. After incorporating a theoretical framework, interviewing participants, and reviewing the literature, 44 items were generated. After a Delphi consultation and a pilot test, 36 items proceeded to item analysis and exploratory factor analysis (EFA), and a four-factor structure with 21 items was formed. Confirmatory factor analysis (CFA), test–retest reliability, criteria-related validity, and internal consistency tests were conducted in phase II to examine the psychometric properties.

**Results:**

There were 21 items on the DCTPFQ, ranging from 1 (fully disagree) through 5 (fully agree). As a result of EFA and CFA, the four factors of DCTPFQ could be verified, including cognitive engagement, subjective experience, medical resources, and relatives and friends’ support. Test–retest reliability of the DCTPFQ was 0.840, and Cronbach’s alpha was 0.934. DCTPFQ is significantly correlated with the Fear of Progression Questionnaire—short form (r = 0.731, *p* < 0.05) and the Mishel’s Uncertainty in Illness Scale (r = 0.714, *p* < 0.05).

**Conclusion:**

The DCTPFQ is a useful tool for measuring the drug clinical trial participation feelings among cancer patients.

## Introduction

A clinical trial refers to an investigation conducted on human subjects with the purpose of examining or confirming the clinical, pharmacological, and other pharmacodynamic evidence of the efficacy of an investigational drug. It also aims to detect any adverse reactions associated with the investigational drug and study its absorption, distribution, metabolism, and excretion of an investigational drug in order to determine its safety and efficacy (NMPA, 2020). According to statistics, the total number of registered clinical trials on the Drug Clinical Trial Registration and Information Disclosure Platform in 2022 exceeded 3,410, representing the highest total annual registration to date. This represents a slight increase compared to the total registration in 2021. In 2022, the majority of the clinical trial projects in China were concentrated in the field of anti-tumor research (Center for drug evaluation, 2021). In clinical trials of anticancer drugs, the majority of participants are patients in advanced stages of malignant tumors that either lack standard treatment options or have experienced treatment failures with standard protocols or those for whom no therapeutic measures are available. Novel anticancer drugs offer new treatment opportunities for these patients. Evidence indicates that among eligible cancer patients meeting the inclusion criteria, over half of them are able to participate in drug clinical trials (Shujun et al., 2022). The public has begun to recognize the benefits of participating in drug clinical trials.

Cancer patients undergoing drug clinical trials usually experience a series of psychological changes, and healthcare professionals are particularly attentive to the psychological shifts and care of these patients both before and after enrollment (Guixia et al., 2018; Shujun et al., 2022). Research has found that cancer patients harbor significant hopes both before and after participation in drug clinical trials (Shaobing and Yingjun, 2008; Caili et al., 2019). Advanced cancer patients anticipate improved treatment outcomes from new drugs, hoping for a cure, and even expecting miracles. However, it is worth noting that some cancer patients also experience fear or apprehension regarding potential adverse reactions of the new drugs being investigated. Patients are the primary contributors and experiencers in drug clinical trials. Investigating the participants’ authentic experiences and perceptions of participating in clinical trials can help understand the reasons behind barriers to active participation and compliance. This information can be instrumental in formulating corresponding strategies to enhance the quality of drug clinical trials.

In 1986, American scholar Meleis introduced the concept of “transition” in medical nursing, referring to the process of shifting from one state, stage, or form to another state, stage, or form (Betz, 1998). Transition period nursing has been widely applied in the care of specific stages of certain diseases, but its content varies for different diseases (Lingjie et al., 2022). For cancer patients, their emotional experiences are complex, and during their participation in drug clinical trials, attention is urgently needed to address feelings of uncertainty about treatment and other related emotions. According to the Roper–Logan–Tierney theory, when a patient’s independence in daily-life activities is altered due to hospitalization, it is influenced by external factors such as the treatment environment, government policies, and the accessibility of welfare (Bellman, 1996; Timmins and O'Shea, 2004a). Cancer patients participating in drug clinical trials constitute a special group that is particularly susceptible to external influences. Negative participant feelings may increase the risk of dropout or loss to follow-up among trial participants (Lixuan et al., 2020). Therefore, it is crucial for medical staff to understand the feelings of cancer patients during their involvement in drug clinical trials. However, currently, there is a lack of an effective assessment tool to measure the participant feelings of cancer patients during drug clinical trials. Thus, this study, based on transition theory and the Roper–Logan–Tierney theory, utilized the Delphi expert consultation method and survey research to develop a questionnaire assessing the participant experiences of cancer patients in drug clinical trials. This study initiative aims to provide a scientifically valid assessment tool and theoretical basis for measuring the participant feelings of cancer patients in drug clinical trials. This study also serves as a reference for clinical medical staff to provide personalized care for cancer patients participating in drug clinical trials in the future.

## Materials and methods

### Study design and subjects

**FIGURE 1:**
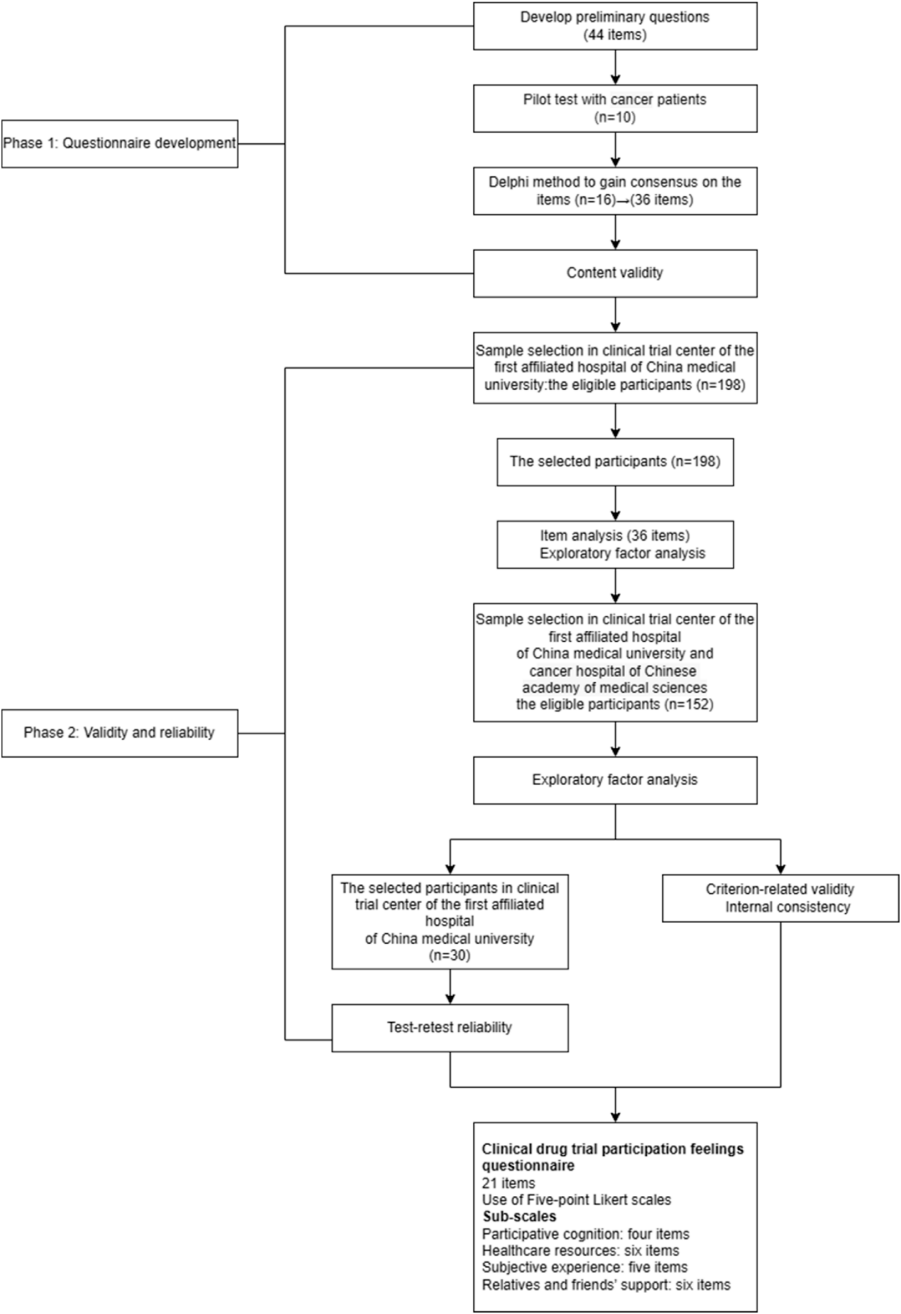
Process of developing and validating a questionnaire.

The study included two phases: questionnaire development and questionnaire validation from March 2022 to September 2023 (Figure 1). Qualitative methods for questionnaire development and quantitative methods for questionnaire validation were used. Ethical approval was obtained from China Medical University’s ethics committee for the study [ethics number: (2022) 98]. We assured the anonymity and confidentiality of the participants throughout the study. The purpose and design of the study, as well as the voluntary nature of participation, were explained to the participants. The participants returned the questionnaire to certify their agreement to participate in this study.

### Questionnaire development

PubMed, Web of Science, and Elsevier databases were searched for “clinical trial,” “drug clinical trial,” “experience,” “feelings,” “psychology,” “neoplasms,” “cancer,” “tumor,” “reliability,” “validity,” “questionnaire,” “scale,” “measure,” “assessment,” “tool,” and “instrument” as the medical subject headings words or different combinations. The retrieval time of the literature studies was from the establishment of the database to 20 April 2022. As an example, the PubMed search strategy is illustrated in Supplementary Appendix 1.

The questionnaire design was based on the transitions theory and the Roper–Logan–Tierney theory. Meleis had developed the transitions theory based on symbolic interactionism and the role theory, which describes the psychosomatic experiences of individuals during a period of new experiences (Meleis, 1975; Meleis, 1997). Meleis delineated four types of transitions, namely, developmental, situational, health/illness, and organizational. In this study, the focus is primarily on situational transitions, and this theory asserts that the person, society, and community serve as the transition conditions. When individuals develop confidence and coping, they can regain a sense of mastery and fluid integrative identities. Assisting individuals in coping with the transition process and facilitating these transitions can meet the evolving needs of individuals and families throughout the course of illness treatment. According to the transitions theory, in the new environment of drug clinical trials, patients strive to redefine their self-concept, urgently desiring to regain a sense of control that they had before, and they require new knowledge and skills to manage their psychophysical states. Based on the transitions theory, cancer patients’ participation experience is primarily influenced by subjective experiences, social customs and culture, socioeconomic status, preparedness and planning, knowledge reserves, and community resources (Meleis, 2010).

**FIGURE 2:**
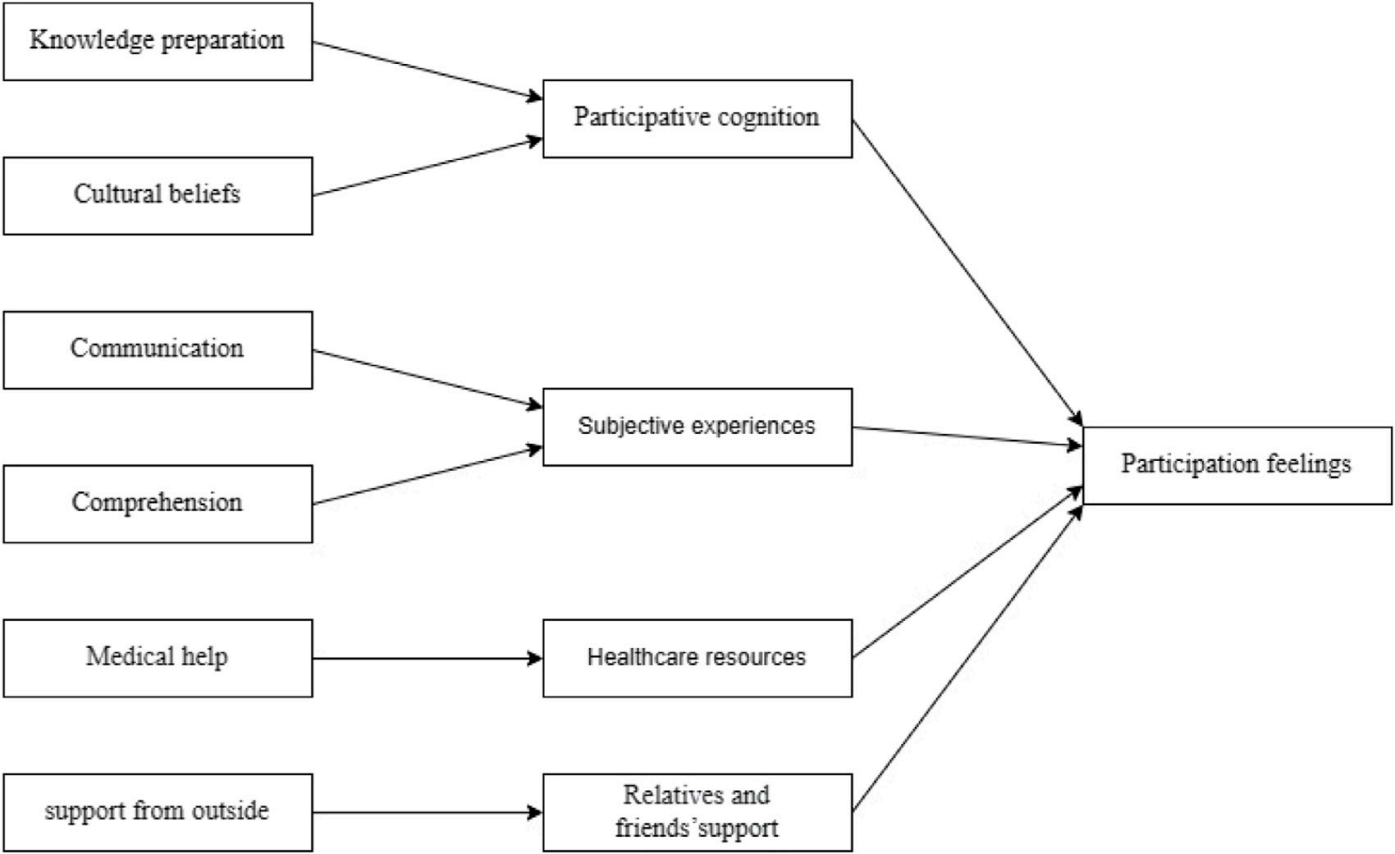
Theoretical framework for this study.

The Roper–Logan–Tierney nursing model is typically used to assess changes in a patient’s independence in daily-life activities due to illness, injury, or hospitalization (Bellman, 1996; Timmins and O'Shea, 2004b). The Roper–Logan–Tierney nursing model considers that factors influencing cancer patients’ drug clinical trial participation experience include their own illness condition, cognitive abilities and understanding, cultural beliefs, external environment, government policies, and accessibility to welfare. Based on the Roper–Logan–Tierney nursing model, this study assesses the changes in the independence of daily-life activities and the current participation experience of cancer patients during their participation in drug clinical trials (Figure 2).

The qualitative study method used in this study is the interview analysis method. By convenience sampling, we collected opinions toward participation feelings of 10 cancer patients from the clinical trial center using a semi-structured and open-ended qualitative interview format. Five of them were male subjects, and five were female subjects, with an age average of (53.00 ± 11.20). The interview focused on knowledge and awareness of clinical drug trials, perceptions and experiences during clinical drug trials, nursing and treatment during clinical drug trials, and care from family and friends of cancer patients. Four aspects of the interview guide are described in the online supplement (see Supplementary Material S1): 1) Participative cognition: disparities in the information sources and knowledge base of cancer patients in drug clinical trials, with questions such as “How much do you know about drug clinical trials?,” “How do you usually acquire drug clinical trial knowledge?,” and “Have you studied any relevant knowledge about drug clinical trials?”. 2) Healthcare resources: medical treatment and care by doctors and nurses for cancer patients during drug clinical trials, with questions such as “What kind of assistance do you hope to receive from doctors and nurses while participating in a drug clinical trial?,” “Can doctors and nurses promptly attend to changes in your condition and your care needs?,” and “Do doctors or nurses use appropriate language and communication methods to fully inform you about the trial drug, the trial’s purpose, the treatment process, potential risks, your rights, and obligations?”. 3) Subjective experience: perceptions and experiences of cancer patients during drug clinical trials, with questions such as “What are your feelings and experiences during your participation in the drug clinical trial?,” “What do you feel you have gained from participating in the clinical trial?,” and “What is your assessment of the drug clinical trial?”. 4) Relatives’ and friends’ support: the care and assistance provided by family and friends to cancer patients during drug clinical trials, with questions such as “Do your family and friends express agreement or support for your participation in the trial?,” “Have your family members been taking care of you throughout your participation in the drug clinical trial?,” and “During your participation in the drug clinical trial, can your family members or friends help you overcome difficulties when you encounter them?.” Each interview lasted 30–40 min.

Based on the transitions theory (Meleis, 2010), the Roper–Logan–Tierney care model (Williams, 2015), and the qualitative interviews of 10 cancer patients participating in a drug clinical trial, a 44-item questionnaire was drafted around four aspects of cognitive engagement, subjective experience, medical resources, and support from family and friends, with a 5-point rating from ‘1’ (fully disagree) to ‘5’ (fully agree). We constructed questions from these domains and ensured that the concept of the questionnaire made sense and the language was understandable.

### Pilot test with cancer patients

We recruited 20 participants for pilot testing before testing the questionnaire’s psychometric properties. Among the 20 participants, the mean age was 59.70 ± 11.69 (range 25–76 years). Pilot testing aims to identify potential questionnaire item miss-phrasing and decide which items need to be modified, added, or deleted. The researchers explained the purpose of the study before its completion for each participant. Minor revisions were made to the wording of the items based on feedback from the participants during the pilot test. The Cronbach’s alpha coefficient was 0.935. In this study, content validity was expressed as the correlation coefficient between the items and the dimensions to which they belonged and the other dimensions. For validity, we found the correlation between each item and its own dimension to be greater than the correlation between the item and the other dimensions. The questionnaire could be considered to have good content validity (Supplementary Appendixes 2, 3, 4, 5). For validity and reliability testing, pilot test participants were excluded to avoid the impact of repeated answers.

### Delphi survey

The Delphi survey was used twice to obtain expert consensus and determine the degree of agreement on the questionnaire. Sixteen experts from the medical field were invited to form an expert consultation group (including psychological specialists and oncology experts) to conduct two rounds of consultation. The expert consultation questionnaire was sent to experts via email. The relevance and importance of each item were assessed by 16 experts on a questionnaire range of 1 (irrelevant) to 4 (extremely relevant) and 1 (unimportant) to 5 (extremely important). The inclusion criteria of experts were as follows: (a) has engaged in psychology and behavior research; (b) has a title of senior level or higher; (c) has a bachelor’s degree or higher; (d) has a working experience of 8 years or above; (e) and has participated in the research on a voluntary basis. Three parts made up the Delphi expert consultation questionnaire: (a) Experts’ general information, including age, working years, educational level, profession title, and research direction. (b) Scoring form for draft clinical drug trials participation feelings questionnaire. Each item was scored based on the Likert five-level scale, and a suggestion column was included based on their expertise and experience. (c) Expert knowledge of the survey’s content and the index’s judgment. In addition, each item on the questionnaire was modified and increased or decreased based on the experts’ professional knowledge and work experience. A preliminary questionnaire with 36 items was modified following two rounds of Delphi surveys since the content validity index (CVI) must be ≥ 0.80 (Yalçın and Baykal, 2019).

The expert’s authority is expressed by a coefficient called expert authority coefficient (Cr). Generally, two factors determine Cr: one is a measure of expert judgment, expressed in Ca, and the other is a measure of an expert’s familiarity with indicators, expressed in Cs (Qiu et al., 2016). As a way of indicating experts’ familiarity, we used a 5-point scale (1.0, 0.8, 0.6, 0.4, and 0.2) ranging from extremely familiar to unfamiliar. The item judgment criteria included practical experience, theoretical analysis of items, knowledge of the literature, and intuitive perceptions. Using a scoring system, the experts’ judgment criteria were rated by the participants (see online Supplementary Material S2), and the participants’ ratings were recorded. When each participant accepted the invitation to participate, informed consent was obtained from them (XH, 2009).

Kendall’s concordance coefficient reflects the degree of coordination between the expert opinions, which is based on the judgment and familiarity coefficients. For high quality, the recovery rate and authority coefficient of the questionnaires must be at least 0.7. Kendall’s test showed a significant result (*p* < 0.05) (Erik et al., 2018).

### Phase 2: validity and reliability

#### Setting and sampling

We calculated the sample size based on Kendall’s principle, which states that the sample size should be five to ten times the number of variables in the study (MacCallum et al., 1999). Based on the consideration of 10% sample loss, the estimated sample size for the present study was 198. We conducted the item analysis and examined a viable factor structure with 198 cancer patients in Shenyang, Liaoning Province, China, between October 2022 and April 2023. All the patients were recruited from the clinical trial center, the First Affiliated Hospital of China Medical University, and the Cancer Hospital Chinese Academy of Medical Sciences. The study population includes cancer patients participating in drug clinical trials for cancer. On this basis, the criterion-related validity and internal consistency of the questionnaire were evaluated. For confirmatory factor analysis, a minimum sample size of 100 is recommended (Bollen, 1989). From May 2023 to September 2023, due to constraints related to the study period and the number of patients presenting with cancer at the clinical trial center, another 152 patients participating in drug clinical trials for cancer patients were selected to verify the suitability of the factor structure for the sample. Informed consent was obtained from all participants prior to completing the survey.

In order to examine questionnaire’s test–retest reliability, data were re-collected after a 2-week interval from 30 cancer patients who were selected from those who had finished the first questionnaire using convenience sampling (Polit and Beck, 2008). The Spearman’s correlation coefficient was used to assess test–retest reliability, and the coefficient ≥ 0.70 indicated acceptable test–retest reliability (Schougaard et al., 2018).

#### Data collection

In the sample survey, the following questions were asked: (1) demographic information such as the gender, age, education level, marital status, residence, occupation, income *per capita*, economic source, whether the tumor has metastasized, whether surgery has been performed, date of tumor diagnosis, and participation in which phases of drug clinical trials. The reasons why, and under what conditions, patients participate in drug clinical trials and the ranking of individuals who have a significant impact on the drug clinical trial were also recorded. (2) There were 36 items in the five-point rating questionnaire for drug clinical trial participation feelings (1 = fully disagree, 2 = disagree, 3 = not sure, 4 = agree, and 5 = fully agree). (3) The Chinese version of Fear of Progression Questionnaire—short form (FoP-Q-SF) (Qiyun et al., 2015), which was mainly proposed by Wu et al. in 2015 to measure the patients’ fear of cancer progression. There are 12 items in the Chinese version of the FoP-Q-SF that cover social/familial aspects and physical health. FoP-Q-SF adopts a five-point scale ranging from 1 (never) to 5 (always). In studies of cancer patients, the Chinese version of the scale has been shown to be reliable and valid (Ban et al., 2021). Higher scores indicate a greater fear of progression. Cronbach’s alpha coefficient of the Chinese version of Fo P-Q-SF was 0.906 in this study. (4) The Chinese version of the Mishel’s Uncertainty in Illness Scale (MUIS) was used to measure the status of the cancer patient’s sense of disease uncertainty (Sheu and Hwang, 1996). The content of the scale included two dimensions of complexity and uncertainty. A total of 25 items are included, with a five-point scale ranging from 1 (strongly disagree) to 5 (strongly agree). Cronbach’s alpha coefficient of the Chinese version of MUIS was 0.931 in this study. A Mandarin description was provided for each item in the survey.

Within 20 min, the investigators guided the cancer patients in filling out the questionnaires anonymously. Investigators collected questionnaires and checked and numbered them one by one. A sample of 152 valid responses of the First Affiliated Hospital of China Medical University, Shenyang, and Cancer Hospital Chinese Academy of Medical Sciences, Beijing, was left, and it reflected a valid response rate of 100%.

#### Data analysis

AMOS V.21.0 (SPSS) and IBM SPSS Statistics V.26.0 (SPSS) were used to analyze the data. IBM SPSS Statistics V.26.0 (SPSS) was used for item analysis, exploratory factor analysis, and reliability validity analysis. AMOS V.21.0 (SPSS) was used for confirmatory factor analysis (CFA). *p* < 0.05 was considered statistically significant. The good–poor analysis, measures of dispersion and Cronbach α coefficient, and the item–total correlations were used for the item analysis. Skewness and kurtosis of study data indicated that they were normally distributed (i.e., skewness less than 2 and kurtosis less than 4) (Wolf and Davis, 2014). Each item’s correlation with the total questionnaire and its criterion-related validity was calculated using a Pearson correlation coefficient. We assessed the data’s suitability for factor analysis using the Kaiser–Meyer–Olkin (KMO) adequacy measure and Bartlett’s test. We conducted an exploratory factor analysis (EFA) and used a promax rotation method for the subsequent estimation. Items with factors loadings of 0.4 or greater were retained, and the factors with an eigenvalue of one or more were extracted (Stevens, 2012). In order to confirm the appropriateness of the constructs in the sample, a confirmatory factor analysis (CFA) was conducted. The cut-off point for the factor loadings was 0.4. We calculated several fit indices, including root-mean-square residuals (RMR), comparative fit indices (CFI), goodness-of-fit indices (GFI), Tucker– Lewis indices (TLI), adjusted goodness-of-fit indices (AGFI), and root-mean-square error of approximation (RMSEA). In order to assess reliability, the internal consistency method was used to calculate Cronbach’s alpha. Internal consistency was calculated using Cronbach’s alpha, with values > 0.7 indicating appropriate internal consistency (Terwee et al., 2007). The reliability of test–retests was used to evaluate stability. The test–retest reliability was considered satisfactory if the correlation coefficient was higher than 0.7 (Noguchi et al., 2016). The validity was evaluated with content validity, construct validity, and criterion-related validity. The McDonald omega is greater than 0.7, and the split-half reliability greater than 0.7 is acceptable (McDonald, 1978; Zhang et al., 2021).

## Results

### Content validity

Combined with the transition theory, the Roper–Logan–Tierney nursing model, and empathy, 44 items were initially designed for the drug clinical trials participation feelings questionnaire. We conducted two rounds of Delphi surveys. According to the results of the first round of Delphi consultation, the Cr was 0.834, while the values of Cs and Ca were 0.800 and 0.866, respectively. Moreover, since 28 items needed to be modified, the research group reviewed each item and added amendments to the next round. There were 38 items in the second round. With most of these items reaching the consensus, the final contents of the questionnaire were determined, and the consultation rounds ended. A total of 36 items were included in the latest questionnaire. The CVI at each item level was over 0.80, and the CVI at the scale-level was 0.97.

### Item analysis

An internal consistency test was performed using Cronbach’s alpha to assess each item and the clinical drug trials participation feelings questionnaire. Correlations between each item’s score and the overall score were examined using the Pearson correlation coefficient. A correlation coefficient of less than 0.40 was removed, and items were removed if they were insignificant (*p* > 0.05) or if they lowered the questionnaire’s Cronbach’s alpha. Three items were deleted because the standard deviation of their standard deviations (item 21, item 24, and item 27) was less than 0.8, which was the threshold for deleting items under the discrete trend method. We repeated the procedure until we were unable to remove any more items. According to the item-level analyses, 15 items were removed. The Cronbach’s alpha of the drug clinical trial participation feelings questionnaire was increased from 0.905 to 0.908. A total of 21 items were further refined as a result of EFA. Details can be found in Supplementary Material S3.

### Construct validity

In the first stage, exploratory factor analysis (EFA) was used to identify the components. A commonly used statistical technique, EFA, was used to remove non-essential variables and determine if there were any associations between them. Screen tests were performed to determine the optimal number of domains to retain using EFA, eigenvalues of chosen components greater than 1, and a percentage of variance explained by all components greater than 50% (UCLA: Statistical Consulting Group, 2019). We used a promax rotation for factor loading and considered questions loading more than 0.3 to contribute to the same domain (J and Pedhazur, 1991). In addition to PCA exploration, exploratory factor analysis (EFA) was conducted on the pilot data (J and Pedhazur, 1991). After the research team discussed and reached a consensus on the statistical relevance of any questions in the pilot data that failed to load onto any domain or loaded onto more than one domain (not statistically relevant), they were removed (Ted et al., 2010).

Based on the Kaiser criterion, there were four factors with factor loadings greater than 0.5 across 21 items of the clinical drug trials participation feelings questionnaire. Four factors were cut off based on eigenvalues greater than 1, and each factor’s loadings were 7.489, 2.074, 1.771, and 1.382, respectively. The principal component method is used to extract the initial common factor, and promax rotation is used to rotate the initial common factor. KMO is a measure of sampling adequacy for the questionnaire, and the Bartlett test of sphericity also reached statistical significance (*p* < 0.001). The correlation matrix’s factor ability was supported by these results. A total of 60.56% of the variance could be explained by the four factors.

EFA identifies four factors, with each factor interpreted based on the items with the highest factor loadings. Factor 1 was labeled as participative cognition as it consisted of four items related to the subject’s perception of the drug clinical trial. Factor 2 comprises six items related to the care and treatment provided by healthcare professionals, which can reflect the resources provided by healthcare personnel. Therefore, factor 2 was labeled as healthcare resources. Factor 3 was labeled as subjective experiences because it included five items related to the subjective experiences of participants during the drug clinical trial. Finally, factor 4 includes six items related to caregiving from family and friends, and this factor was identified as relatives and friends’ support. There was a correlation between the item-total of 0.504–0.644, and all *p*-values were less than 0.05. In Table 1, the loadings and factor structure of the items, percentage variance explained by each factor, and item–total correlations are presented.

**TABLE 1:** Exploratory factor analysis of clinical drug trial participation feelings questionnaire in cancer patients.

Item	Factor loadings				h2	Item–total correlation
	1	2	3	4		
Participative cognition						
1				0.810	0.659	0.570
6				0.719	0.523	0.504
2				0.717	0.547	0.556
7				0.656	0.450	0.513
Healthcare resources						
13		0.795			0.650	0.559
16		0.823			0.686	0.644
15		0.777			0.646	0.546
12		0.794			0.633	0.636
9		0.786			0.640	0.605
14		0.761			0.609	0.613
Subjective experiences						
18			0.772		0.615	0.527
22			0.788		0.625	0.588
19			0.750		0.573	0.508
20			0.743		0.565	0.538
23			0.582		0.368	0.525
Relatives and friends’ support						
33	0.841				0.711	0.627
30	0.832				0.697	0.586
34	0.814				0.666	0.551
31	0.803				0.646	0.566
32	0.793				0.651	0.604
35	0.725				0.556	0.577
Eigenvalue	7.489	2.074	1.771	1.382		
Percentage of variance explained (%)	35.663	9.878	8.434	6.582		
Cumulative percentage of variance explained (%)	35.663	45.541	53.975	60.556		

**FIGURE 3:**
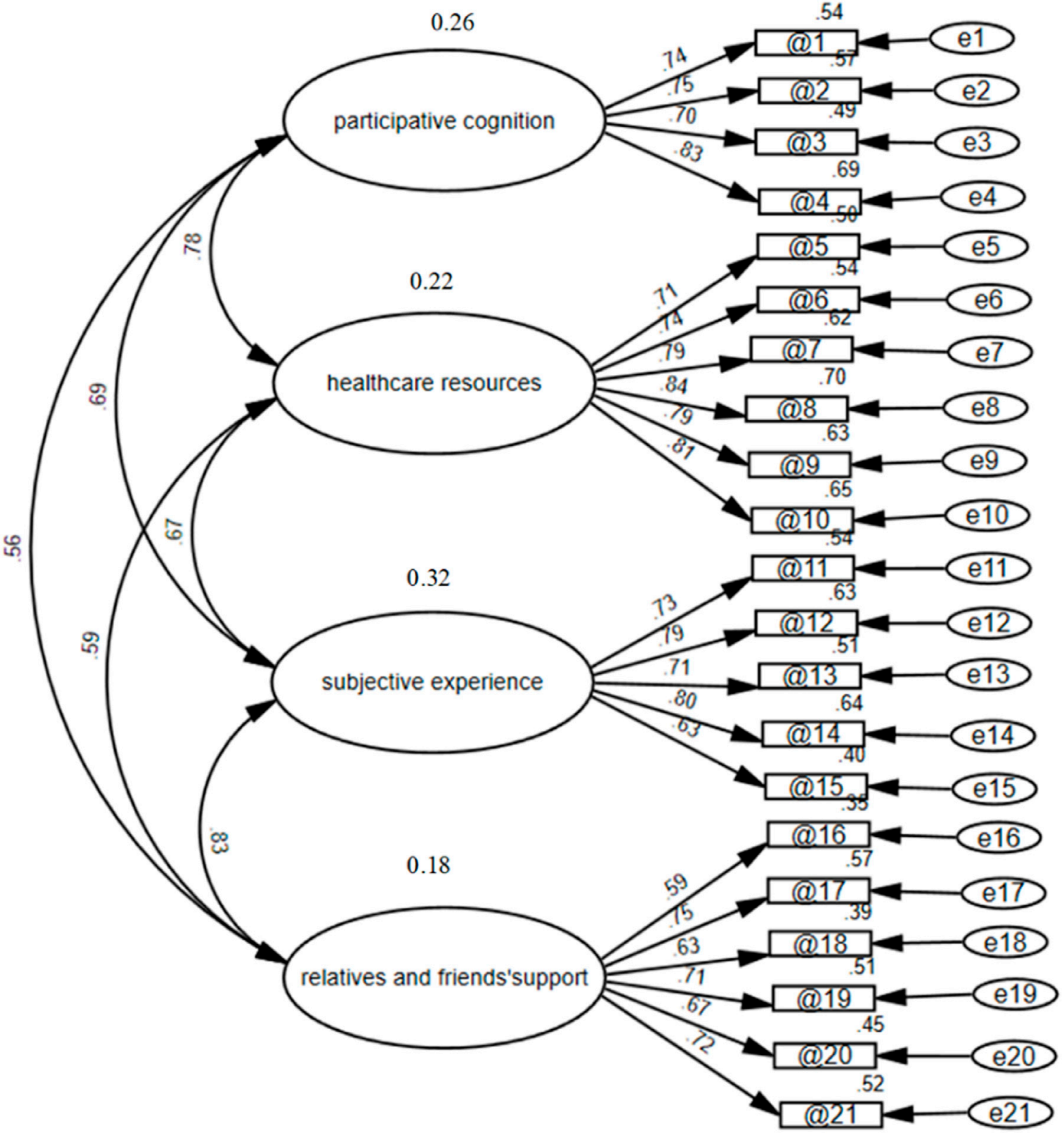
Confirmatory factor analysis of the clinical drug trials participation feelings questionnaire in cancer patients participating in drug clinical trials.

The correlated four-factor measurement model developed based on EFA was validated using confirmatory factor analysis (CFA). The structure of the questionnaire was validated by confirmatory factor analysis (CFA). In Figure 3, the final factor structure model of the CFA is presented. The model-fitting results showed that the standardized coefficient for each path ranged from 0.59 to 084, all above 0.4, which was above the acceptable standard. The final model revealed a good fit (Doll et al., 1994) to the data (χ2/df = 1.175, RMR = 0.022, CFI = 0.980, GFI = 0.885, TLI = 0.978, AGFI = 0.855, and RMSEA = 0.034). Using this analysis, we can identify four distinct dimensions correlated with the questionnaire as a second-order construct. The fitting index reached the acceptable standard of the model, indicating that the data fit of the structural equation model was good, suggesting that the questionnaire had good construct validity.

### Criterion-related validity

We also conducted a correlation analysis to compare the clinical drug trials participation feelings questionnaire with the FoP-Q-SF and MUIS to confirm the criterion-related validity of the clinical drug trials participation feelings questionnaire. Table 2 shows the calculated correlation coefficients that confirm a good criterion-related validity of the clinical drug trial participation feelings questionnaire for FoP-Q-SF and MUIS.

**TABLE 2:** Correlation coefficients between the drug clinical trial participation feelings questionnaire and FoP-Q-SF and MUIS for cancer patients.

Factor	MUIS	FoP-Q-SF
Participative cognition	0.587*	0.608*
Healthcare resources	0.598*	0.585*
Subjective experience	0.606*	0.629*
Relatives and friends’ support	0.584*	0.612*
DCTPFQ	0.714*	0.731*

*p < 0.01.

MUIS, the Chinese version of Mishel’s Uncertainty in Illness Scale. DCTPFQ, Drug clinical trial participation feelings questionnaire

FoP-Q-SF, the Chinese version of the Fear of Progression Questionnaire—short form.

### Reliability

Internal consistency (Cronbach’s alphas) and test–retest reliability were used to calculate the questionnaire’s reliability. Cronbach’s alpha for the questionnaire was 0.934, and its Cronbach’s alphas for each factor were 0.840, 0.903, 0.854, and 0.837, respectively. The test–retest reliability values of the four factors were 0.819, 0.750, 0.778, and 0.791. Test–retest reliability was 0.840 for the questionnaire (*p* < 0.01). We report the reliability of the questionnaire using McDonald’s omega reliability coefficients and Guttmann split-half reliability. The McDonald omega was 0.934, and the split-half reliability was 0.801.

## Discussion

Drug clinical trials, as one of the optimal or alternative treatment options for advanced cancer patients, require each participant to fully comprehend the potential benefits and risks associated with participating in a new drug clinical trial and understand the inevitability of these risks. Given the risks and public welfare nature of clinical trials for cancer drugs, a comprehensive and thorough understanding of the trial by cancer patients during the drug clinical trial process forms the foundation of their participation feelings (Yue et al., 2013). The participation feelings of cancer patients during drug clinical trials significantly influence the treatment outcome (Tingting et al., 2023). Moreover, the participation feelings of cancer patients are an indispensable factor in driving the development of clinical trials for cancer drugs. Studies on the psychological experiences of cancer patients participating in drug clinical trials have increased in recent years (Yihong et al., 2023). However, there is a shortage of systematic assessment tools for the overall participation feelings in clinical trials for cancer drugs. For clinical trials of cancer drugs, some aspects of participant feelings are unknown, exploratory, or exceed initial expectations. Hence, a comprehensive assessment tool should be developed to assess cancer patients’ feelings in drug clinical trials.

In this study, our data were collected from cancer patients participating in drug clinical trials to facilitate the development of the DCTPFQ. In the initial questionnaire, patients participating in cancer clinical trials gave their advice and opinions along with experts in the related fields. Thereafter, the topic expression and items were gradually revised until the final DCTPFQ included 21 items across four factors (please refer to Supplementary Material S5 for English and Supplementary Material S6 for Chinese). The final version of the questionnaire yielded resulted to show appropriate confirmed content, internal consistency reliability, construct validity, and criterion validity.

In this study, the Fear of Progression Questionnaire—short form—and Mishel’s Uncertainty in Illness Scale with high reliability and validity were used as criterial-related indicators. The correlation coefficients all reached the significance level of 0.05, indicating that there was a positive correlation between the questionnaire and other measurement instruments. The participation feelings of cancer patients was related to the fear of illness progression and uncertainty in illness, which was consistent with the research of Qian et al. (2024), indicating that the criterion-related validity of the questionnaire was ideal. The correlation coefficient reflects the linear correlations between instruments but does not fully account for their measured uniqueness, although r = 0.7 indicates that 50% of the variance is common. There is a clear theoretical distinction between the constructs we measured in our study and the constructs of other measurement instruments. Meleis’ transitions theory and the Roper–Logan–Tierney theory are important theoretical frameworks in the medical field, aimed at understanding and supporting patients’ adaptation processes during various transitional periods (Meleis, 1975; Bellman, 1996). The transitions theory and the Roper–Logan–Tierney theory particularly emphasize assisting patients in effectively coping with changes and transitions in clinical practice. Based on the transition theory and the Roper–Logan–Tierney theory, the questionnaire developed in this study explores patients’ feelings during clinical trials from four aspects: cognitive engagement, subjective experience, medical resources, and relatives and friends’ support. However, the Fear of Progression Questionnaire—short form—and Mishel’s Uncertainty in Illness Scale demonstrate unique attributes and functions in assessing the psychological state of fear of disease progression and the psychological feeling of uncertainty during the illness process (Sheu and Hwang, 1996; Qiyun et al., 2015).

In the final version of the questionnaire, four domains were included: “participative cognition,” “healthcare resources,” “subjective experience,” and “relatives and friends’ support”. The four factors matched empirical evidence with the transitions theory and the Roper–Logan–Tierney nursing model (Timmins and O'Shea, 2004a; Meleis, 1975). During clinical trials of cancer drugs, cancer patients actively search information about drug clinical trials, utilizing their cognition and comprehension abilities to develop participative cognition. Healthcare resources, including diagnosis, treatment, and care provided by doctors and nurses, along with relatives’ and friends’ support, contribute to the subjective experiences of cancer patients in relation to drug clinical trials, thereby fostering the formation of participation feelings.

Participative cognition, the first factor of the questionnaire, refers to an individual’s level of cognitive engagement in a process, encompassing their understanding and involvement in the process (Wenjing et al., 2022). A previous study pointed out that participative cognition has an influential impact on the psychological well-being of cancer patients during drug clinical trials (Guangju et al., 2022). Participative cognition is associated with information gathering (Kirkpatrick et al., 2023), interpersonal trust with members of the clinical trial team (Hurd et al., 2017), and self-efficaciousness (Gouveia et al., 2022). For example, item 3, “I am prepared to deal with any challenges that may arise during the drug clinical trial,” reflects the participation feelings that cancer patients perceive from affirming their own abilities and interpersonal trust with the clinical trial team. In drug clinical trials, encountering challenges is inevitable. Only through active communication with the drug clinical trial team and sharing any concerns, discomfort, or issues can one address personal troubles and ensure receiving appropriate assistance.

The participative cognition factor of a questionnaire is mainly concerned with the cancer patients’ feelings during the drug clinical trial from the aspects of personal preparation, interpersonal trust, and self-efficaciousness. Through these factors, the participative cognition level of cancer patients can be precisely identified to help medical staff conduct targeted nursing and ultimately improve participative cognition. High participative cognition contribute to a better understanding of drug clinical trials, fostering a greater sense of their own involvement, and enhancing the participation feelings for cancer patients during drug clinical trials.

Healthcare resources is one of the essential elements in shaping the participation feelings of cancer patients during drug clinical trials, which involves the assistance and support that clinical research medical staff provide to their patients (Portier, 2020; Park and Yu, 2022). Clinical research medical staff possesses solid professional knowledge and skills regarding disease treatment and care. Simultaneously, medical staff is equipped with abilities in education, coordination, and management (Xiaohong et al., 2021). They are responsible for tasks such as patient assessment, education, symptom observation, monitoring adverse reactions, and follow-up during clinical care. Based on the self-regulation theory, individuals consciously monitor their behavior and continuously adjust their emotions and thoughts based on expectations (Kuhl et al., 2006). However, without medical staff actively engaging with cancer patients in drug clinical trials, patients may develop negative feelings about their participation (McKinney et al., 2021; Shiely et al., 2023). Therefore, ensuring adequate healthcare resources for cancer patients is an effective way to promote the development of positive participation feelings.

With the emphasis on healthcare resources in various clinical trials, researchers have paid increasing attention to the training of clinical trial teams’ nursing ability (Lavender and Croudass, 2019). Previous psychological experience research on drug clinical trials for participants also included healthcare resources as an imperative aspect (Canidate et al., 2020). In the healthcare resources factor, our questionnaire mainly examines the cancer patients’ participation feelings of communicating with medical staff and nursing. For instance, item 6, “During my participation in the drug clinical trial, the medical staff communicated with me thoroughly,” and item 10, “During my participation in the drug clinical trial, I will consult with the medical staff about questions related to diagnosis, treatment, and care,” indicate the cancer patients’ participation feelings of consulting medical staff during the drug clinical trial. During the drug clinical trial, the communication and medical care provided by the medical staff have effectively assisted them to understand the clinical trial, fostering positive participation feelings among cancer patients.

Subjective experience is the third factor of the DCTPFQ, which affects patients’ fit with the clinical trial team and affects the overall performance (Nishtar et al., 2023). In other words, the more positive the subjective experience of cancer patients, the more readily they endorse drug clinical trials, and the easier it is to maintain positive participation feelings. A study on the development of questionnaires for drug clinical trials also found that subjective experience is a crucial factor in measuring the feelings of cancer patients during the trial (Le et al., 2022). Therefore, in this factor, content that can foster the formation of positive participation feelings is included.

Within the factor of subjective experience, a few items, such as item 12, “I believe that the drug clinical trial will have a positive impact on the treatment of my condition,” and item 14, “From the beginning of participating in the drug clinical trial until now, I have been actively and proactively involved throughout,” revealed the cancer patients’ participation feelings of both clinical treatment and personal involvement. By reviewing this content, cancer patients can cultivate positive expectations for drug clinical trials, adjust their mindset, or enhance their personal involvement to develop a positive perspective on drug clinical trials (Wray et al., 2007; Schilling et al., 2019). The continuous adjustment of expectations and personal involvement is not only the result of subjective experience but also a method proposed in this study to foster the positive participation feelings in drug clinical trials.

Relatives’ and friends’ support, the fourth factor of the questionnaire, refers to the assistance provided by family and friends to the patients in their daily lives, including both material and emotional support (Bloom et al., 2023; Narayanan et al., 2023). The importance of support from family and friends lies not only in alleviating physical ailments but also in establishing a robust social support system, aiding patients in better facing various challenges (Xiaojing and Rui, 2021). Research suggests a strong correlation between having a supportive network of family and social connections and the physical recovery of patients, as well as a higher level of drug clinical trial satisfaction (Xiaohui et al., 2019). The item “During my participation in the drug clinical trial, I received excellent care from my family” provided a good evaluation for the care of family members in clinical trials. Therefore, when designing medical services and formulating treatment plans during drug clinical trials, the roles of family members and friends should be taken into consideration. Measures should be implemented to encourage and facilitate their active involvement in the participation of cancer patients during drug clinical trials. This comprehensive support system is expected to have a positive and profound impact on the participation feelings of cancer patients and the quality of drug clinical trials.

This study also has some limitations. Drug clinical trial participation feelings are not only influenced by external support but also by individual differences. Consequently, the questionnaire may not be able to accommodate different life backgrounds when exploring the participation feelings. The questionnaire also takes into account the current situation of cancer patients’ age ratio in China; in other words, the majority of the population consists of middle-aged and elderly people. It may be necessary to examine the research results further if the ratio of middle-aged and elderly people to young changes. The sample size was limited by the number of cancer patients of the drug clinical trial in the two hospitals and the research cycle. Lastly, when applied to patients with different diseases, this questionnaire needs to be developed and evaluated further for its universal adaptability. Researchers’ assessment of cancer patients’ feelings of participation can help make up for the deficiencies of traditional nursing measures in drug clinical trials. Knowing patients’ participation feelings in drug clinical trials can provide substantial support and suggestions for improving patient participation, optimizing the clinical trial treatment process, and improving doctor–patient communication.

## Conclusion

The participation feelings of cancer patients are an essential factor in the field of drug clinical trials. As a result of this study, we developed a tool for measuring cancer patients’ feelings about their participation in drug clinical trials. In this study, the DCTPFQ for cancer patients was divided into four dimensions: participative cognition, healthcare resources, subjective experience, and relatives and friends’ support. This study provided sufficient evidence for the reliability and validity of DCTPFQ in evaluating the participation feelings of cancer patients. Therefore, researchers involved in drug clinical trials should use DCTPFQ or apply it to the participation feelings evaluation of cancer patients, contributing to the drug clinical trial research of cancer patients.

## Data Availability

The original contributions presented in the study are included in the article/Supplementary Material; further inquiries can be directed to the corresponding authors.
