# Supplementary material for: Development and validation of a drug clinical trial participation feelings questionnaire for cancer patients
Source: Front Pharmacol. 2024 Jun 18;15:1371811. doi: 10.3389/fphar.2024.1371811 (PMC11217336; doi:10.3389/fphar.2024.1371811)
Supplement: Supplementary file 2 [file DataSheet2.docx]

**Appendix**

Appendix 1 PubMed search strategy, 20 April 2022

| Set | Search terms |
| --- | --- |
| #1 | clinical trial [MeSH Terms] |
| #2 | drug clinical trial [All Fields] |
| #3 | #1 OR #2 |
| #4 | experience [All Fields] |
| #5 | feelings [All Fields] |
| #6 | psychology [All Fields] |
| #7 | #4 OR #5 OR #6 |
| #8 | neoplasms [MeSH Terms] |
| #9 | cancer [Title/Abstract] |
| #10 | tumor [Title/Abstract] |
| #11 | #8 OR #9 OR #10 |
| #12 | reliability [All Fields] |
| #13 | validity [All Fields] |
| #14 | #12 OR #13 |
| #15 | questionnaire [All Fields] |
| #16 | scale [All Fields] |
| #17 | measure [All Fields] |
| #18 | assessment [All Fields] |
| #19 | tool [All Fields] |
| #20 | instrument [All Fields] |
| #21 | #15 OR #16 OR #17 OR #18 OR #19 OR #20 |
| #22 | Search (#3 AND #7 AND #11 AND #14 AND #21) |

Appendix 2 Results of the correlation analysis between the cognitive engagement items and the dimensions of the drug clinical trial participation feelings questionnaire（r_s_）

| Dimensions | V1 | V2 | V3 | V4 | V5 | V6 | V7 |
| --- | --- | --- | --- | --- | --- | --- | --- |
| Cognitive engagement | 0.715^**^ | 0.758^**^ | 0.632^**^ | 0.825^**^ | 0.516^*^ | 0.582^**^ | 0.825^**^ |
| Subjective experience | 0.647^**^ | 0.607^**^ | 0.179 | 0.615^**^ | 0.167 | 0.364 | 0.789^**^ |
| Medical resources | 0.689^**^ | 0.501* | 0.331 | 0.708^**^ | 0.379 | 0.476^*^ | 0.772^**^ |
| Relatives and friends’ support | 0.669^**^ | 0.538^*^ | 0.242 | 0.580^**^ | 0.309 | 0.365 | 0.764^**^ |

*：P＜0.05；**：P＜0.01；

Appendix 3 Results of the correlation analysis between the medical resources items and the dimensions of the drug clinical trial participation feelings questionnaire（r_s_）

| Dimensions | V8 | V9 | V10 | V11 | V12 | V13 | V14 | V15 | V16 | V24 |
| --- | --- | --- | --- | --- | --- | --- | --- | --- | --- | --- |
| Cognitive engagement | 0.743^**^ | 0.153 | 0.311 | 0.302 | 0.380 | 0.564^**^ | 0.388 | 0.649^**^ | 0.649^**^ | 0.632^**^ |
| Subjective experience | 0.535^*^ | 0.170 | 0.361 | 0.264 | 0.280 | 0.678^**^ | 0.447^*^ | 0.523^*^ | 0.523^*^ | 0.527^*^ |
| Medical resources | 0.594^**^ | 0.616^**^ | 0.739^**^ | 0.642^**^ | 0.543* | 0.700^**^ | 0.652^**^ | 0.789^**^ | 0.789^**^ | 0.723^**^ |
| Relatives and friends’ support | 0.594^**^ | 0.260 | 0.256 | 0.106 | 0.509* | 0.582^**^ | 0.421 | 0.515^*^ | 0.515^*^ | 0.439 |

*：P＜0.05；**：P＜0.01；

Appendix 4 Results of the correlation analysis between the subjective experience items and the dimensions of the drug clinical trial participation feelings questionnaire（r_s_）

| Dimensions | V17 | V18 | V19 | V20 | V21 | V22 | V23 | V25 | V26 | V27 | V28 |
| --- | --- | --- | --- | --- | --- | --- | --- | --- | --- | --- | --- |
| Cognitive engagement | 0.311 | 0.180 | 0.386 | 0.501^*^ | 0.370 | 0.286 | 0.347 | 0.019 | 0.248 | 0.320 | 0.525^*^ |
| Subjective experience | 0.710^**^ | 0.534^*^ | 0.790^**^ | 0.704^**^ | 0.445^*^ | 0.564^**^ | 0.463^*^ | 0.447^*^ | 0.680^**^ | 0.473^*^ | 0.596^**^ |
| Medical resources | 0.106 | 0.231 | 0.248 | 0.459^*^ | 0.313 | 0.202 | 0.333 | -0.038 | 0.175 | -0.026 | 0.167 |
| Relatives and friends’ support | 0.485^*^ | 0.330 | 0.307 | 0.359 | 0.188 | 0.274 | 0.456^*^ | 0.010 | 0.325 | 0.229 | 0.609^*^ |

*：P＜0.05；**：P＜0.01；

Appendix 5 Results of the correlation analysis between the relatives and friends’ support items and the dimensions of the drug clinical trial participation feelings questionnaire（r_s_）

| Dimensions | V29 | V30 | V31 | V32 | V33 | V34 | V35 | V36 |
| --- | --- | --- | --- | --- | --- | --- | --- | --- |
| Cognitive engagement | 0.524^*^ | 0.631^*^ | 0.190 | 0.408 | 0.613^**^ | 0.579^**^ | 0.738^**^ | 0.382 |
| Subjective experience | 0.321 | 0.384 | 0.258 | 0.119 | 0.666^**^ | 0.409 | 0.719^**^ | 0.307 |
| Medical resources | 0.554^*^ | 0.495^*^ | 0.018 | 0.303 | 0.422 | 0.544^*^ | 0.422 | 0.193 |
| Relatives and friends’ support | 0.724^**^ | 0.725^**^ | 0.616^**^ | 0.551^*^ | 0.695^**^ | 0.630^**^ | 0.757^**^ | 0.642^**^ |

*：P＜0.05；**：P＜0.01

| Appendix 6 Test of data normality | | | | | |  |
| --- | --- | --- | --- | --- | --- | --- |
| Items | Skewness  coefficient | Kurtosis  coefficient | Items | Skewness  coefficient | Kurtosis  coefficient |  |
| 1 | -0.785 | -0.114 | 12 | -0.591 | -0.744 |  |
| 2 | -0.444 | -0.894 | 13 | -0.585 | -0.540 |  |
| 3 | -0.445 | -0.587 | 14 | -0.610 | -0.759 |  |
| 4 | -0.401 | -0.844 | 15 | -0.589 | -0.801 |  |
| 5 | -1.150 | 0.088 | 16 | -0.943 | -0.075 |  |
| 6 | -0.792 | -0.544 | 17 | -0.701 | -0.402 |  |
| 7 | -0.787 | -0.618 | 18 | -0.342 | -0.845 |  |
| 8 | -0.840 | -0.695 | 19 | -0.873 | -0.213 |  |
| 9 | -0.880 | -0.338 | 20 | -0.475 | -0.969 |  |
| 10 | -0.835 | -0.495 | 21 | -1.175 | 0.778 | |
| 11 | -0.686 | -0.712 |  |  |  |  |

| Appendix 7 Demographic characteristics of the participants (n=152) | | |
| --- | --- | --- |
| Variable | Number | Constituent ratio(%) |
| Gender |  |  |
| Male | 89 | 58.60 |
| Female | 63 | 41.40 |
| Participation in which phases of drug clinical trial |  |  |
| Ⅰ | 74 | 48.68 |
| Ⅱ | 42 | 27.63 |
| Ⅲ | 32 | 21.05 |
| Ⅳ | 2 | 1.32 |
| Other | 2 | 1.32 |
| Whether surgery has been performed |  |  |
| Yes | 87 | 57.24 |
| No | 65 | 42.76 |
| Whether the tumor has metastasized |  |  |
| Yes | 93 | 61.18 |
| No | 59 | 38.82 |
| Marital status |  |  |
| With a spouse | 136 | 89.47 |
| Without a spouse | 15 | 9.87 |
| Other | 1 | 0.66 |
| Residence |  |  |
| Urban | 129 | 84.90 |
| Rural areas | 23 | 15.10 |
| Occupation |  |  |
| Retire | 91 | 59.87 |
| Employed by another person or organisation | 18 | 11.84 |
| Farming | 15 | 9.87 |
| Jobless | 12 | 7.89 |
| Temporary work (no formal labour contract) | 9 | 5.92 |
| Running a self-employed or private business | 7 | 4.60 |
| Income per capita, yuan |  |  |
| ≤2000 | 41 | 26.97 |
| 2001~4999 | 61 | 40.13 |
| ≥5000 | 50 | 32.89 |
| Economic source |  |  |
| Payroll | 32 | 21.05 |
| Retirement or pension | 91 | 59.87 |
| Farming | 15 | 9.87 |
| Partial allowance for children | 5 | 3.29 |
| Minimum subsistence allowance | 3 | 1.97 |
| Investors | 1 | 0.66 |
| Other | 5 | 3.29 |
| The reasons why, and under what conditions, patients participate in drug clinical trial |  |  |
| Recommended to me by healthcare professionals | 135 | 70.68 |
| Self-understanding during hospitalisation | 23 | 12.04 |
| Acquired in the course of social life | 12 | 6.28 |
| Known through some information on the internet and other media | 8 | 4.19 |
| Recommended by a non-medical relative or friend | 7 | 3.66 |
| Recommended by a relative or friend in the medical profession | 6 | 3.14 |

Note: Certain constituent ratio do not equal 100 due to rounding.
