# Supplementary material for: Development and validation of a drug clinical trial participation feelings questionnaire for cancer patients
Source: Front Pharmacol. 2024 Jun 18;15:1371811. doi: 10.3389/fphar.2024.1371811 (PMC11217336; doi:10.3389/fphar.2024.1371811)
Supplement: Supplementary file 3 [file DataSheet1.docx]

**Supplementary Files**

**Supplementary File 1 In-depth interview outline of cancer patients**

Dear fellow patients:

Thank you for your participation. I would like to interview you about some problems in clinical drug trials participation feelings to learn the current status of knowledge and awareness of clinical drug trials, perceptions and experiences during clinical drug trials, nursing and treatment during clinical drug trials, and care from family and friends of cancer patients to explore the real participation feelings of cancer patients. This interview would last for about 30-40 minutes with high confidentiality.

1. **Disparities in the information sources and knowledge base of cancer patients in clinical drug trials:**

- How much do you know about drug clinical trials?
- Have you studied any relevant knowledge about drug clinical trials?
- How do you usually acquire drug clinical trials knowledge?

1. **Medical treatment and care by doctors and nurses for cancer patients during clinical drug trials:**

- What kind of assistance do you hope to receive from doctors and nurses while participating in a clinical drug trial?
- Can doctors and nurses promptly attend to changes in your condition and your care needs?
- Do doctors or nurses use appropriate language and communication methods to fully inform you about the trial drug, the trial’s purpose, the treatment process, potential risks, your rights, and obligations?

**(3) Perceptions and experiences of cancer patients during clinical drug trials:**

- What are your feelings and experiences during your participation in the drug clinical trial?
- What do you feel you have gained from participating in the clinical trial?
- What is your assessment of the drug clinical trial?

**(4) The care and assistance provided by family and friends to cancer patients during clinical drug trials：**

- Do your family and friends express agreement or support for your participation in the trial?
- Have your family members been taking care of you throughout your participation in the drug clinical trial?
- During your participation in the drug clinical trial, can your family members or friends help you overcome difficulties when you encounter them?

**Supplementary File 2** Criterion for judgment and scoring system

| Judgment Criterion | The Degree of Impact on Experts' Judgement | | |
| --- | --- | --- | --- |
|  | Large Impact | Medium Impact | Medium Impact |
| Experience | 0.50 | 0.40 | 0.30 |
| Theoretical analysis | 0.30 | 0.20 | 0.10 |
| Knowledge of literature | 0.10 | 0.08 | 0.05 |
| Instinct | 0.10 | 0.07 | 0.05 |

**Supplementary File 3** Item analysis of the drug clinical trial participation feelings questionnaire in cancer patients

| Item | Cronbach’s  alpha | Good-poor  Analysis | Item-total correlation (r) | P-values | Exclusion |
| --- | --- | --- | --- | --- | --- |
| 1 | 0.901 | <0.05 | 0.570 | <0.05 | 🗸 |
| 2 | 0.902 | <0.05 | 0.556 | <0.05 | 🗸 |
| 3 | 0.903 | <0.05 | 0.460 | <0.05 | 🗸 |
| 4 | 0.903 | <0.05 | 0.434 | <0.05 | 🗸 |
| 5 | 0.903 | <0.05 | 0.445 | <0.05 | 🗸 |
| 6 | 0.902 | <0.05 | 0.504 | <0.05 | 🗸 |
| 7 | 0.902 | <0.05 | 0.513 | <0.05 | 🗸 |
| 8 | 0.903 | <0.05 | 0.453 | <0.05 | 🗸 |
| 9 | 0.901 | <0.05 | 0.605 | <0.05 | 🗸 |
| 10 | 0.903 | <0.05 | 0.499 | <0.05 | 🗸 |
| 11 | **0.907** | **0.110** | **0.148** | <0.05 | × |
| 12 | 0.900 | <0.05 | 0.636 | <0.05 | 🗸 |
| 13 | 0.902 | <0.05 | 0.559 | <0.05 | 🗸 |
| 14 | 0.901 | <0.05 | 0.613 | <0.05 | 🗸 |
| 15 | 0.902 | <0.05 | 0.546 | <0.05 | 🗸 |
| 16 | 0.900 | <0.05 | 0.644 | <0.05 | 🗸 |
| 17 | 0.904 | <0.05 | 0.400 | <0.05 | 🗸 |
| 18 | 0.902 | <0.05 | 0.527 | <0.05 | 🗸 |
| 19 | 0.902 | <0.05 | 0.508 | <0.05 | 🗸 |
| 20 | 0.902 | <0.05 | 0.538 | <0.05 | 🗸 |
| 21 | 0.903 | <0.05 | 0.482 | <0.05 | 🗸 |
| 22 | 0.901 | <0.05 | 0.588 | <0.05 | 🗸 |
| 23 | 0.902 | <0.05 | 0.525 | <0.05 | 🗸 |
| 24 | **0.906** | <0.05 | **0.234** | <0.05 | × |
| 25 | **0.907** | <0.05 | **0.257** | <0.05 | × |
| 26 | 0.903 | <0.05 | 0.435 | <0.05 | 🗸 |
| 27 | **0.905** | <0.05 | **0.295** | <0.05 | × |
| 28 | **0.905** | <0.05 | **0.366** | <0.05 | × |
| 29 | **0.905** | <0.05 | **0.363** | <0.05 | × |
| 30 | 0.901 | <0.05 | 0.586 | <0.05 | 🗸 |
| 31 | 0.902 | <0.05 | 0.566 | <0.05 | 🗸 |
| 32 | 0.901 | <0.05 | 0.604 | <0.05 | 🗸 |
| 33 | 0.901 | <0.05 | 0.627 | <0.05 | 🗸 |
| 34 | 0.902 | <0.05 | 0.551 | <0.05 | 🗸 |
| 35 | 0.901 | <0.05 | 0.577 | <0.05 | 🗸 |
| 36 | **0.908** | <0.05 | **0.257** | <0.05 | × |

Exclusion criteria for the item analyses: The values which fit the exclusion criteria were written in bold.

1. The Cronbach's alpha of each item was smaller than the overall alpha of the questionnaire.
2. The significance test of the difference between the high and low groups in the average score of each item. P≤0.05 indicated a significant difference.
3. The correlation coefficient between the item and the total score of all the items: less than 0.4. P≤0.05 indicated a significant difference.

(4) "🗸" indicated the items that had been reserved, while "×" indicated excluded by using a reverse scoring.


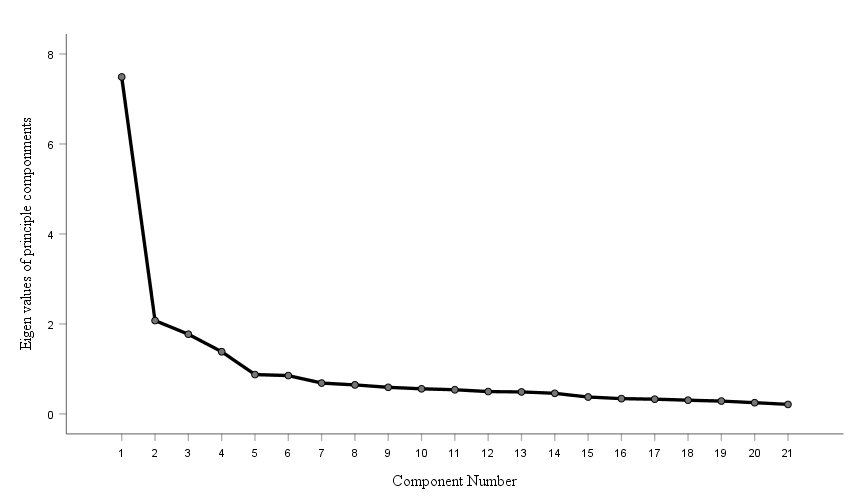


**Supplementary File 4** A four-factor solution of EFA was used based upon the results ofthe eigenvalues of principle components

**Supplementary File 5** The Drug Clinical Trial Participation Feelings Questionnaire (English version)

|  | Item | fully agree | agree | not sure | disagree | fully disagree |
| --- | --- | --- | --- | --- | --- | --- |
| Participative cognition | 1.I believe in the standardization and safety of drug clinical trial | 5 | 4 | 3 | 2 | 1 |
|  | 2.I believe that the benefits of participating in drug clinical trial outweigh the drawbacks | 5 | 4 | 3 | 2 | 1 |
|  | 3.I am prepared to deal with any challenges that may arise during the drug clinical trial | 5 | 4 | 3 | 2 | 1 |
|  | 4.I believe I can adapt to the drug clinical trial | 5 | 4 | 3 | 2 | 1 |
| Healthcare resources | 5.During my participation in the drug clinical trial, I trust that the medical staff will consider things from my perspective | 5 | 4 | 3 | 2 | 1 |
|  | 6.During my participation in the drug clinical trial, the medical staff communicated with me thoroughly | 5 | 4 | 3 | 2 | 1 |
|  | 7.During my participation in the drug clinical trial, I will receive encouragement from the medical staff involved in the trial | 5 | 4 | 3 | 2 | 1 |
|  | 8.The medical staff will assist me in resolving any issues that may arise during my participation in the drug clinical trial | 5 | 4 | 3 | 2 | 1 |
|  | 9.During my participation in the drug clinical trial, I received excellent care from the medical staff | 5 | 4 | 3 | 2 | 1 |
|  | 10.During my participation in the drug clinical trial, I will consult with the medical staff about questions related to diagnosis, treatment, and care | 5 | 4 | 3 | 2 | 1 |
| Subjective experiences | 11.I believe I can successfully complete the drug clinical trial | 5 | 4 | 3 | 2 | 1 |
|  | 12. I believe that the drug clinical trial will have a positive impact on the treatment of my condition | 5 | 4 | 3 | 2 | 1 |
|  | 13.During my participation in the drug clinical trial, I understand my emotional state | 5 | 4 | 3 | 2 | 1 |
|  | 14.From the beginning of participating in the drug clinical trial until now, I have been actively and proactively involved throughout | 5 | 4 | 3 | 2 | 1 |
|  | 15.I have sufficient time to participate in the drug clinical trial | 5 | 4 | 3 | 2 | 1 |
| Relatives and friends’support | 16.During my participation in the drug clinical trial, I have consistently felt the assistance and support from the organization | 5 | 4 | 3 | 2 | 1 |
|  | 17.When I encounter problems, I can seek help from family and friends | 5 | 4 | 3 | 2 | 1 |
|  | 18.My family or friends frequently listen earnestly to my participation feelings of the drug clinical trial | 5 | 4 | 3 | 2 | 1 |
|  | 19.My family members support my participation in drug clinical trial | 5 | 4 | 3 | 2 | 1 |
|  | 20.My family understands my feelings during the drug clinical trial | 5 | 4 | 3 | 2 | 1 |
|  | 21.During my participation in the drug clinical trial, I received excellent care from my family | 5 | 4 | 3 | 2 | 1 |

**Supplementary File 6** The Drug Clinical Trials Participation Feelings Questionnaire (Chinese version)

药物临床试验参与感受问卷

|  | 题目 | 非常同意 | 同意 | 不确定 | 不同意 | 非常不同意 |
| --- | --- | --- | --- | --- | --- | --- |
| 参与认知 | 1.我相信药物临床试验的规范性与安全性 | 5 | 4 | 3 | 2 | 1 |
|  | 2.我认为参与药物临床试验利大于弊 | 5 | 4 | 3 | 2 | 1 |
|  | 3.我已经做好准备去应对药物临床试验期间可能遇到的问题 | 5 | 4 | 3 | 2 | 1 |
|  | 4.我相信自己能适应药物临床试验 | 5 | 4 | 3 | 2 | 1 |
| 医护资源 | 5.参与药物临床试验期间，医护人员能站在我的角度考虑问题 | 5 | 4 | 3 | 2 | 1 |
|  | 6.参与药物临床试验期间，医护人员能够与我充分交流 | 5 | 4 | 3 | 2 | 1 |
|  | 7.参与药物临床试验期间，我会得到药物临床试验医护人员的鼓励 | 5 | 4 | 3 | 2 | 1 |
|  | 8.医护人员会帮我解决参与药物临床试验期间遇到的问题 | 5 | 4 | 3 | 2 | 1 |
|  | 9.参与药物临床试验期间，我得到了医护人员良好的照护 | 5 | 4 | 3 | 2 | 1 |
|  | 10.参与药物临床试验期间，我会向医护人员咨询诊疗与护理方面的问题 | 5 | 4 | 3 | 2 | 1 |
| 主观体验 | 11.我相信自己能顺利完成药物临床试验 | 5 | 4 | 3 | 2 | 1 |
|  | 12.我认为药物临床试验对我的疾病治疗会产生积极作用 | 5 | 4 | 3 | 2 | 1 |
|  | 13.参与药物临床试验期间，我了解自己的情绪状态 | 5 | 4 | 3 | 2 | 1 |
|  | 14.从开始参加药物临床试验至今，我一直积极、主动地参与其中 | 5 | 4 | 3 | 2 | 1 |
|  | 15.我有足够的时间参与药物临床试验 | 5 | 4 | 3 | 2 | 1 |
| 亲友支持 | 16.参与药物临床试验期间，我始终能感知到组织机构的帮助 | 5 | 4 | 3 | 2 | 1 |
|  | 17.当我遇到问题时我可以寻求亲人和朋友的帮助 | 5 | 4 | 3 | 2 | 1 |
|  | 18.亲人或朋友经常认真倾听我参与药物临床试验的感受 | 5 | 4 | 3 | 2 | 1 |
|  | 19.我的家庭成员支持我参与药物临床试验 | 5 | 4 | 3 | 2 | 1 |
|  | 20.家人能理解我参与药物临床试验期间的感受 | 5 | 4 | 3 | 2 | 1 |
|  | 21.参与药物临床试验期间，我得到家人很好的照顾 | 5 | 4 | 3 | 2 | 1 |
